# A Simpler Creatinine Index Can Predict Long-Term Survival in Chinese Hemodialysis Patients

**DOI:** 10.1371/journal.pone.0165164

**Published:** 2016-10-25

**Authors:** Chiung-Ying Huang, Szu-Ying Lee, Chung-Wei Yang, Szu-Chun Hung, Chih-Kang Chiang, Jenq-Wen Huang, Kuan-Yu Hung

**Affiliations:** 1 Department of Internal Medicine, National Taiwan University College of Medicine and Hospital, Taipei City, Taiwan; 2 Department of Internal Medicine, National Taiwan University College of Medicine and Hospital, Yun-Lin Branch, Yun-Lin County, Taiwan; 3 Department of Internal Medicine, National Taiwan University College of Medicine and Hospital, Hsin-Chu Branch, Hsin Chu City, Taiwan; 4 Division of Nephrology, Taipei Tzu Chi Hospital, New Taipei City, Taiwan; Chi-Mei Medical Center, TAIWAN

## Abstract

**Background:**

Low lean body mass (LBM) is an indicator of malnutrition inflammation syndrome, which is common in hemodialysis (HD) patients. The creatinine index (CI) has been validated as a reliable method to estimate LBM and evaluate the protein-energy status of HD patients. However, the traditional creatinine index formula was complex. We sought to investigate the impact of CI derived from a new simple formula on Chinese HD patient outcomes.

**Methods:**

In this retrospective cohort study, we enrolled 1269 patients who initiated HD between February 1981 and February 2012 and followed them until the end of February 2013. CI was calculated using the simple creatinine kinetic model (CKM) formula. Multiple linear regression analysis and Cox regression proportional hazard analysis were used to define independent variables and compare survival between groups.

**Results:**

The 1269 HD patients were categorized into 3 groups according to the tertiles of calculated CI between men and women. Each group consisted of 423 patients (50.6% men, 49.4% women). Patients in the highest sex-specific tertile of CI had longer overall survival (HR, 0.46; P 0.002). BMI did not significantly associate with survival after adjustment (HR,0.99; P 0.613).

**Conclusions:**

CI derived from the simple CKM formula serves as a good parameter than BMI to predict the survival of HD patients. The formula could extend its convenient use in clinical practice for HD patients.

## Introduction

A high body mass index (BMI) has been reported to be predictive of lower mortality in hemodialysis (HD) patients, but the relative importance of body fat and lean body mass (LBM) is unclear[1]. However, a recent study found that the excess weight in HD patients is related to fat and extracellular water and an inverse correlation exists between BMI and LBM[2]. Low LBM is an indicator of protein-energy wasting and malnutrition inflammation syndrome; therefore, it significantly correlates with higher mortality in dialysis patients[3, 4].

Accurate measurement of LBM is important to evaluate nutritional status and predict prognosis in dialysis patients. Many methods have been used to measure LBM, such as dual energy X-ray absorptiometry (DEXA), bioimpedance analysis (BIA), and anthropometric measurement, but they are not readily accessible in clinical practice.

Estimation of LBM using the creatinine index(CI) derived from traditional creatinine kinetic modeling (CKM) has been validated as a reliable method for muscle mass assessment and correlates significantly with LBM measured by BIA in dialysis patients [5]. In our previous study, LBM measured by CI was a good predictor of long-term survival in Chinese patients on peritoneal dialysis [6]. The creatinine index was also a good predictor of survival in French HD patients[7]. However, the traditional CI formula is complex and requires postdialysis serum creatinine concentration and dialysate and urine collection, which are not routinely measured in HD patients. Therefore, the use of CI is limited in clinical practice for HD patients.

Recently, a simpler formula that uses patient demographics, single pool Kt/V of urea (spKt/V), and predialysis serum creatinine concentration was developed and validated for calculating CI in HD patients[8], but the clinical value of this parameter is not yet clear. The aim of this study was to investigate the clinical significance of CI derived from this formula on HD patient outcomes.

## Methods

### Subjects

**Fig 1:**
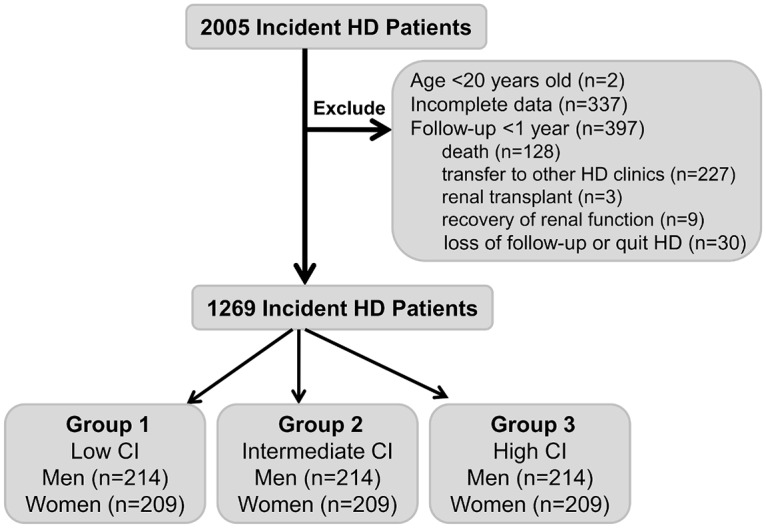
Flow diagram detailing the inclusion and exclusion of patients in this study.

In this retrospective cohort study, 2005 patients who initiated HD as chronic renal replacement therapy between February 1981 and February 2012 from 3 HD centers in Taiwan were initially enrolled. Patients were excluded if they were <20 years of age (n = 2), had a follow-up period of <1 year (n = 397), or had incomplete baseline characteristic or laboratory data (n = 337). The characteristics of patients who had a follow-up period less than 1 year were reviewed. There were 128 patients died; 227 patients transferred to other HD clinics; 3 patients received renal transplantation; 9 patients became hemodialysis independent; 30 patients lost of follow-up or quit of hemodialysis. After these exclusions, there were 1269 patients enrolled in the study (Fig 1). Follow-up continued until the end of February 2013.

### Clinical characteristics and follow-up

Clinical characteristics and dialysis parameters, including BMI, spKt/V, and normalized protein catabolic rate (nPCR), were obtained from medical records. The results of regular serum chemistry studies, including hemoglobin, blood urea nitrogen (BUN), creatinine, albumin, calcium-phosphorous product, total cholesterol, triglycerides, and total iron binding capacity (TIBC), were also recorded. These data were collected 1 month after HD initiation. CI was calculated using the simple creatinine index formula. Because some patients received HD twice a week and some received HD thrice a week, we used weekly Kt/V to represent the HD clearance. The nPCR was calculated using the following formula[9]:

Beginning of week nPCR:

$${\text{BUN}}_{\text{pre}}/\left(36.3\;+\;\left[5.48\right]\left[\text{spKt}/\text{V}\right]+\;\left[\left(53.5\right)/\left(\text{spKt}/\text{V}\right)\right]\right)\;+0.168$$

Midweek nPCR:

$${\text{BUN}}_{\text{pre}}/\left(25.8\;+\;\left[1.15\right]\left[\text{spKt}/\text{V}\right]+\;\left[\left(56.4\right)/\left(\text{spKt}/\text{V}\right)\right]\right)\;+0.168$$

End of week nPCR:

$${\text{BUN}}_{\text{pre}}/\left(16.3+\;\left[4.3\right]\left[s\text{pKt}/\text{V}\right]+\;\left[\left(56.6\right)/\left(\text{spKt}/\text{V}\right)\right]\right)\;+0.168$$

After initiation of HD, patients were followed prospectively for the occurrence of mortality. Patients who received transplants or transferred to other HD clinics were censored at time of transplant or transfer.

### Ethical considerations

All medical records and laboratory data of the 2005 patients were reviewed in this study. The study was approved by the ethics committee of the National Taiwan University Hospital (NTUH) under NTUH-REC No. 201303005RINC. The institutional review board did not mandate patient consent, since patient record was de-identified prior to analysis, and patient privacy was not breached.

### Calculation of creatinine index and lean body mass

The simple formula for CI prediction calculated from dialysis dose and patient demographics was developed via mixed regression models from measured CKM-derived CI. The mixed regression model was [8]:

$$\text{CI (mg/kg/day) }=\;16.21+1.12\times [1\text{ if male; }0\text{ if female}]-0.06\times \text{age }\left(\text{years}\right)-0.08\times \text{spKt/Vurea}+\;0.009\times {\text{Cr}}_{\text{pre}}\text{(μmol/L)}$$

### Statistical analysis

All variables are reported as mean *±*SD (or with 95% confidence intervals where appropriate) for continuous variables and as frequencies or percentages for categorical variables. ANOVA was used for analysis between groups where appropriate. Differences in frequency were tested using Chi square analysis. Relationships between variables were tested using Pearson correlation. The independent determinants of any variable were analyzed using multiple linear regression analysis. The adjusted variables were stated in each analysis. Kaplan–Meier survival analysis and Cox regression proportional hazard analysis were used to analyze survival rates between groups and predictors for survival, respectively. P values <0.05 were considered significant. The statistical analyses were performed using SPSS 19.0 for Windows (SPSS Inc., IL, USA).

## Results

### CI in patients on HD

The 1269 HD patients were categorized into 3 groups according to the tertiles of calculated CI between men and women. Each group consisted of 423 patients (50.6% men, 49.4% women). The patients in group 1 had the lowest CI, group 2 had an intermediate CI, and group 3 had the highest CI. Baseline demographic, clinical, and laboratory values were compared among the 3 groups and are shown in Table 1. Compared with group 3 and group 2 patients, group 1 patients were older; had a higher incidence of diabetes mellitus (DM); had lower BMI and nPCR; and had lower serum levels of albumin, hemoglobin, calcium-phosphorus (CaxP) product, BUN, creatinine, cholesterol, triglycerides, and TIBC. The HD clearance was not significantly different between these 3 groups.

**Table 1:** Comparison of baseline demographic, clinical, and laboratory parameters between HD patients with low CI (group 1), intermediate CI (group 2), and high CI (group 3).

	Group 1	Group 2	Group 3	P value
Age*(y)	71 ± 10	62 ± 10	52 ± 13	<0.001
** Men**	69 ± 11	61 ± 10	52 ± 12	<0.001
** Women**	72 ± 10	64 ± 10	52 ± 13	<0.001
**Men (%)**	50.6	50.6	50.6	1
**DM** *(%)	59	51	28	<0.001
**Hemoglobin** *(g/dL)	9.6 ± 1.4	10.0 ± 1.3	10.1 ± 1.4	<0.001
CaxP*	38.9 ± 13.3	47.2 ± 16.4	55.2 ± 16.9	<0.001
BUN* **(mg/dL)**	63.2 ± 18.6	74.5 ± 20.9	82.3 ± 19.4	<0.001
Creatinine* **(mg/dL)**	6.9 ± 1.4	9.5 ± 1.2	12.4 ± 2.0	<0.001
**Albumin** *(g/dL)	3.5 ± 0.5	3.7 ± 0.4	3.9 ± 0.4	<0.001
**Cholesterol** * **(mg/dL)**	159 ± 38	172 ± 44	172 ± 42	<0.001
Triglycerides* **(mg/dL)**	142 ± 113	157 ± 124	183 ± 182	<0.001
TIBC*	211 ± 45	224 ± 46	229 ± 46	<0.001
**nPCR** *(g/kg BW/day)	0.98 ± 0.28	1.14 ± 0.30	1.21 ± 0.29	<0.001
**Weekly Kt/V**	3.87 ± 0.95	4.0 ± 0.86	3.96 ± 0.90	0.1
**BMI** *(kg/m^**2**^**)**	22.0 ± 3.7	22.9 ± 3.7	23.1 ± 3.9	<0.001
Creatinine index*	17.9 ± 1.5	20.5 ± 1.4	23.4 ± 2.1	<0.001
** Men**	19.0 ± 1.2	21.7 ± 0.7	24.8 ± 1.5	<0.001
** Women**	16.9 ± 1.0	19.3 ± 0.6	21.9 ±1.4	<0.001

*P<0.05 using ANOVA test for continuous variables or the Chi-square test for categorical variables.

BUN, blood urea nitrogen; BMI, body mass index; CaxP, calcium-phosphorus product; DM, diabetes mellitus; nPCR, normalized protein catabolic rat; TIBC, total iron binding capacity.

### Predictors of CI

The relationship between CI and other nutritional and clinical parameters was analyzed using Pearson correlation. CI negatively correlated with age and HD clearance and positively correlated with hemoglobin, CaxP product, BUN, creatinine, triglycerides, TIBC, and albumin levels as well as BMI and nPCR (Table 2). Total cholesterol levels were irrelevant to CI.

**Table 2:** Correlation between CI and other clinical characteristics in all HD patients.

	r	P value
Age*	−0.61	<0.001
Hemoglobin*	0.21	<0.001
Albumin*	0.33	<0.001
**Cholesterol**	0.02	0.59
Triglyceride*	0.09	0.002
BUN*	0.36	<0.001
Creatinine*	0.95	<0.001
CaxP*	0.39	<0.001
Weekly Kt/V*	−0.15	<0.001
**nPCR** *	0.25	<0.001
TIBC*	0.19	<0.001
BMI*	0.15	<0.001

*P<0.05 using Pearson correlation.

BUN, blood urea nitrogen; BMI, body mass index; CI, creatinine index; nPCR, normalized protein catabolic rate; TIBC: total iron binding capacity; r, correlation coefficient.

Independent determinants for CI were further analyzed using multiple linear regression analysis. Hemoglobin, albumin, triglycerides, CaxP product, nPCR, and BMI were positively associated and age, female sex, and history of DM were negatively associated with CI independently (Table 3). These independent determinants contributed to a high predictability (R^2^ = 0.69; Table 3).

**Table 3:** Independent predictors for CI with multiple linear regression analysis among HD patients.

	B ± SD	P value
**Constant**	19.91 ± 0.63	<0.001
**Age**	−0.10 ± 0.004	<0.001
**Women**	−2.29 ± 0.10	<0.001
**DM**	−0.90 ± 0.10	<0.001
**Hemoglobin**	0.16 ± 0.03	<0.001
**Albumin**	0.50 ± 0.11	<0.001
**Triglycerides (per 10 mg/dL)**	0.02 ± 0.003	<0.001
**CaxP**	0.03 ± 0.003	<0.001
**nPCR**	1.68 ± 0.16	<0.001
**BMI**	0.07 ± 0.01	<0.001
**R**^**2**^	0.69	

BMI, body mass index; CI, creatinine index; CaxP, calcium-phosphorus index; DM, diabetes mellitus; nPCR, normalized protein catabolic rate.

### CI and patient outcomes

**Fig 2:**
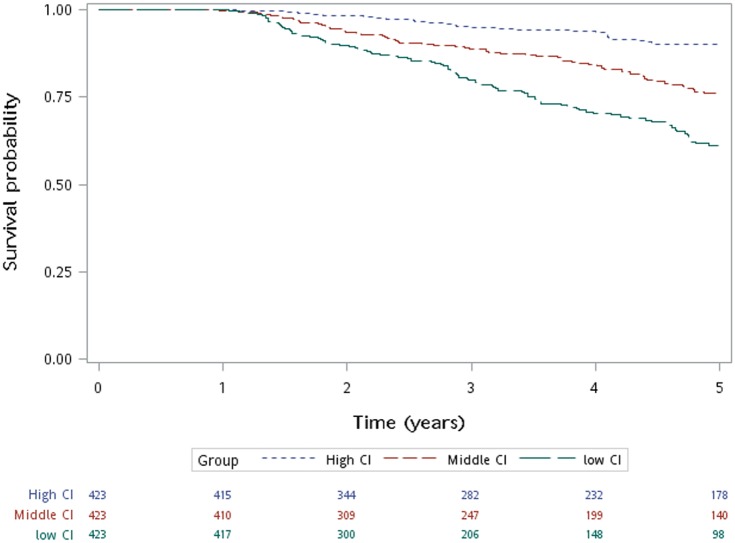
Creatinine Index and Survival. Patients with a lower CI had shorter survival than those with a higher CI according to Kaplan-Meier survival analysis. (P<0.001).

By the end of the follow-up period of this study, 282 (22.2%) patients had died. Kaplan–Meier survival analysis was performed to compare survival among groups. Patients with the highest tertile of CI in group 3 had longer survival (Fig 2, P< 0.001).

Cox regression proportional hazard analysis was applied to determine the hazard ratio (HR) for mortality. CI is a good prognostic marker. Patients with the highest tertile of CI in group 3 had the best survival (Model 1; Table 4). This was true after adjusting for age, DM, Gender, Hb, CaxP product, BUN, Albumin, Cholesterol, Triglycerides, TIBC, nPCR, Weekly Kt/V, and BMI (Model 2; Table 4). BMI did not significantly associate with survival after adjustment (HR,0.99; P 0.613). In subgroup analysis, the inverse relationship of CI and mortality also existed in male HD patients but not female (Table 5).

**Table 4:** Hazard ratio for mortality according to Cox regression proportional hazard analysis in all HD patients.

Mortality			
**Model 1**	**HR**	**95% CI for HR**	**P value**
Creatinine index group			
Creatinine index group (2)	0.53	0.41–0.70	<0.001
Creatinine index group (3)	0.26	0.19–0.36	<0.001
**Model 2**	**HR**	**95% CI for HR**	**P value**
Age	1.03	1.02–1.05	<0.001
DM	1.84	1.37–2.48	<0.001
Hemoglobin (g/dl)	1.04	0.93–1.15	0.517
CaxP	1.01	1.001–1.02	0.037
BUN (mg/dl)	1.01	0.99–1.02	0.53
Albumin (g/dl)	0.78	0.54–1.11	0.165
Cholesterol (mg/dl)	0.996	0.99–1.00	0.053
Triglycerides (mg/dl)	1.00	1.00–1.001	0.328
TIBC (per 10 mg/dL)	0.97	0.94–0.99	0.045
nPCR(g/kg BW/day)	0.80	0.25–2.52	0.699
Weekly Kt/V	1.05	0.87–1.27	0.612
BMI (kg/m^2^)	0.99	0.95–1.03	0.613
Creatinine index group			0.003
Creatinine index group (2)	0.64	0.45–0.91	0.012
Creatinine index group (3)	0.46	0.28–0.75	0.002

BUN, blood urea nitrogen; BMI, body mass index; nPCR, normalized protein catabolic rat; CI, confidence interval; CaxP, calcium-phosphorus product; DM, diabetes mellitus; HR, hazard ratio; TIBC, total iron binding capacity.

**Table 5:** Hazard ratio for mortality according to Cox regression proportional hazard analysis in male and female patients.

Mortality			
**Men**	**HR**	**95% CI for HR**	**P value**
Age	1.02	1.003–1.04	0.02
DM	2.14	1.42–3.22	<0.001
Hemoglobin (g/dl)	1.05	0.91–1.21	0.49
CaxP	1.02	1.002–1.03	0.02
BUN (mg/dl)	1.003	0.98–1.03	0.79
Albumin (g/dl)	0.57	0.35–0.92	0.02
Cholesterol (mg/dl)	0.998	0.99–1.003	0.43
Triglycerides (mg/dl)	1.00	0.99–1.001	0.92
TIBC (per 10 mg/dL)	0.97	0.93–1.01	0.10
nPCR (g/kg BW/day)	1.75	0.31–10.04	0.53
Weekly Kt/V	1.15	0.86–1.54	0.35
BMI (kg/m^2^)	1.02	0.95–1.08	0.62
Creatinine index group			
Creatinine index group (2)	0.56	0.34–0.90	0.018
Creatinine index group (3)	0.41	0.22–0.79	0.008
**Women**			
Age	1.04	1.02–1.06	0.001
DM	1.53	0.98–2.40	0.06
Hemoglobin (g/dl)	0.98	0.84–1.14	0.75
CaxP	1.004	0.99–1.02	0.58
BUN (mg/dl)	0.99	0.98–1.02	0.93
Albumin (g/dl)	1.01	0.56–1.79	0.98
Cholesterol (mg/dl)	0.996	0.99–1.002	0.17
Triglycerides (mg/dl)	1.001	0.99–1.002	0.22
TIBC (per 10 mg/dL)	0.97	0.92–1.02	0.16
nPCR (g/kg BW/day)	0.73	0.14–3.79	0.71
Weekly Kt/V	0.99	0.76–1.31	0.98
BMI (kg/m^2^)	0.97	0.91–1.03	0.31
Creatinine index group			
Creatinine index group (2)	0.79	0.47–1.34	0.38
Creatinine index group (3)	0.47	0.21–1.01	0.053

BUN, blood urea nitrogen; BMI, body mass index; nPCR, normalized protein catabolic rat; CI, confidence interval; CaxP, calcium-phosphorus product; DM, diabetes mellitus; HR, hazard ratio.

## Discussion

According to the analysis of the Taiwan national dialysis registry, the cumulative survival rate of dialysis patients in the 1990–1999 incident cohort at 1 year was 85.1%[10]. In our study, the 1-year survival rate was 73.4%, including those died within the first 1 month of dialysis. In our study, the patients were recruited in hospital based dialysis centers, therefore more patients initiated short-term hemodialysis due to critical condition and died within 1 year. In order to investigate the clinical significance of creatinine index on chronic HD patients and followed up the long-term effect of creatinine index, we selected only those patients who had been receiving HD for more than 1 year to exclude patients who is not long term dialysis dependent and whose life expectancy less than 1 year due to other critical condition. The study showed that a higher CI which derived from the simpler formula predicts longer survival in chronic HD patients. This adds further support to the conclusion that the simpler CI calculation could predict Chinese HD patient outcomes.

Previous study showed that high BMI is associated with increased survival in patients on dialysis, but this association cannot be applied to Asian patients on dialysis[11]. One may speculate that the risk associated with obesity might outweigh the nutritional benefits in Asian patients in contrast to non-Asian patients[12]. In addition, excess weight in HD patients is accompanied by inflammation and inversely associated with LBM[2]. Overhydration is prevalent and can contribute to excess weight in HD patients, and it is associated with a higher mortality rate. Therefore, BMI or body weight is not a good prognostic marker. In our study, BMI did not significantly associate with survival either (HR,0.99; P 0.613). On the other hand, body composition might be more closely associated with prognosis in HD patients. Some studies revealed that a low LBM predicts higher mortality in dialysis patients[6, 13–15]. However, it is difficult to measure LBM accurately. CKM has been validated as a reliable method to estimate LBM and assess protein-energy status in dialysis patients[5, 6, 8].

Both DEXA and BIA might not detect malnutrition in overhydrated dialysis patients due to overestimation of LBM, but CKM, which measures the true muscle degradation production, could reflect the true LBM even in edematous conditions[9, 16]. The simplified equation for CI calculation developed by Canaud, *et al*.closely correlated with CKM-derived CI (correlation coefficient = 0.79, P < 0.001)[8]. This equation can provide a simple method to estimate CI in HD patients. In our study, the simple CI equation was applied in a Chinese HD population, and we found that higher CI had lower mortality, an effect that persisted after adjustment (Table 4), whereas higher CI appears associated with longer survival in men but not in women in sub-group analysis (Table 5). These results support the conclusion that this simple CI equation is a useful tool to predict Chinese HD patient survival independently, especially in men.

Serum albumin level is a well-known indicator of visceral protein, and it is inversely associated with mortality in HD patients[17, 18]. However, the mortality risks associated with serum albumin were not only a consequence of malnutrition, but also inflammation[19]. Serum albumin level can be influenced by inflammation and nutritional status, so it might be insensitive. CKM-derived LBM represents somatic protein storage and is a good tool for assessing protein-energy status; however, the relationship between CI and albumin level remains unclear. In our study, CI was positively correlated with serum albumin levels after adjustment in the HD population.

Protein-energy wasting is highly prevalent in HD patients[13]. LBM is commonly used for nutritional assessment and inversely correlates with mortality in this population [4, 20]. In previous studies, CI from creatinine kinetics correlated significantly with LBM form bioimpedance technique and the simple CI equation closely correlated with CKM-derived CI[5, 8]. In this study, CI could predict survival in HD patients. CI can be calculated conveniently from this simple equation in HD patients because all variables in the equation are measured regularly in clinical practice. The influence of overhydration and obesity are lower in this method than in other measurements. Clinicians could calculate serial CIs from this simplified CI equation in each HD patient, and the change of CI over time could be used as a nutritional and prognostic monitoring tool in dialysis practice.

This was a multicenter study, and it is the first study using this simplified CI equation to predict the outcome in HD patients. There are potential limitations in this observational study. Patients usually initiate HD with a glomerular filtration rate <5 mL/min/1.73m^2^ in Taiwan according to health insurance regulations. Since residual renal function is uncommon among HD patients, we do not have this data and may have underestimated the CI of HD patients with residual renal function. However, although CI may have been underestimated, patients with a higher CI still had lower mortality than those with a lower CI. Finally, as in other studies, we cannot account for unmeasured or residual confounding variables.

## Conclusion

Low CI is a strong predictor of mortality in HD patients. Although this equation needs to be validated by further studies, the simplified CI equation provides a quick and easy method to assess protein-energy status serially in HD patients. This parameter can also serve as a good predictor of Chinese HD patient survival.

## Supporting Information

S1 data The raw data of patients in this study. Clinical characteristics, dialysis parameters, and the results of serum chemistry studies which collected 1 month after HD initiation.
(XLS)
